# Apolipoprotein D Transgenic Mice Develop Hepatic Steatosis through Activation of PPARγ and Fatty Acid Uptake

**DOI:** 10.1371/journal.pone.0130230

**Published:** 2015-06-17

**Authors:** Marilyne Labrie, Simon Lalonde, Ouafa Najyb, Maxime Thiery, Caroline Daneault, Chrisitne Des Rosiers, Eric Rassart, Catherine Mounier

**Affiliations:** 1 Centre de recherche BioMed, Département des Sciences Biologiques, Université du Québec, Montréal, Québec, H3C 3P8, Canada; 2 Department of Nutrition, Université de Montréal, Montréal, Québec, H3C 3J7,Canada; 3 Montreal Heart Institute Research Center, Montreal, Quebec, H1T 1C8,Canada; University of Basque Country, SPAIN

## Abstract

Transgenic mice (Tg) overexpressing human apolipoprotein D (H-apoD) in the brain are resistant to neurodegeneration. Despite the use of a neuron-specific promoter to generate the Tg mice, they expressed significant levels of H-apoD in both plasma and liver and they slowly develop hepatic steatosis and insulin resistance. We show here that hepatic PPARγ expression in Tg mice is increased by 2-fold compared to wild type (WT) mice. Consequently, PPARγ target genes Plin2 and Cide A/C are overexpressed, leading to increased lipid droplets formation. Expression of the fatty acid transporter CD36, another PPARgamma target, is also increased in Tg mice associated with elevated fatty acid uptake as measured in primary hepatocytes. Elevated expression of AMPK in the liver of Tg leads to phosphorylation of acetyl CoA carboxylase, indicating a decreased activity of the enzyme. Fatty acid synthase expression is also induced but the hepatic lipogenesis measured in vivo is not significantly different between WT and Tg mice. In addition, expression of carnitine palmitoyl transferase 1, the rate-limiting enzyme of beta-oxidation, is slightly upregulated. Finally, we show that overexpressing H-apoD in HepG2 cells in presence of arachidonic acid (AA), the main apoD ligand, increases the transcriptional activity of PPARγ. Supporting the role of apoD in AA transport, we observed enrichment in hepatic AA and a decrease in plasmatic AA concentration. Taken together, our results demonstrate that the hepatic steatosis observed in apoD Tg mice is a consequence of increased PPARγ transcriptional activity by AA leading to increased fatty acid uptake by the liver.

## Introduction

Apolipoprotein D (apoD), a 29 kDa glycoprotein, is a member of the lipocalin super family [1]. It transports several small hydrophobic compounds such as arachidonic acid (AA), progesterone, pregnenolone, bilirubin, cholesterol and E-3-methyl-2-hexenoic acid [2–7]. In human, apoD is found in the plasma fraction, associated with high-density lipoprotein (HDL). It is highly expressed in the brain, adrenal glands, kidneys, pancreas and placenta and to a lower extent in intestine and liver [1,8–10]. In contrast, the murine expression of the apoD gene is almost exclusively expressed in the central nervous system (CNS) [11,12].

We have previously shown that transgenic mice (Tg) overexpressing human apoD (H-apoD) in the brain are protected against neurodegeneration and injuries [13,14] suggesting that apoD could be a good therapeutic target against neurodegenerative diseases. Unfortunately, these mice develop, with age, insulin resistance, glucose intolerance as well as hepatic and muscular steatosis [15].

Our previous observations showed that the peroxisome proliferator-activated gamma (PPARγ) mRNA expression is increased in the liver of H-apoD Tg mice [15]. PPARγ is a nuclear receptor implicated in adipocyte differentiation. Two isoforms exist: PPARγ1 is ubiquitously expressed while PPARγ2 is almost exclusively expressed in the adipose tissue [16,17]. When activated by one of its ligands, PPARγ heterodimerizes with retinoid X receptor α (RXRα) and binds to the peroxisome proliferator response elements (PPRE) on the promoter of its target genes [18,19]. PPARγ regulates positively its own transcription and induces transcription of the CCAAT/enhancer-binding protein α (C/EBPα), which in turn also activates PPARγ gene expression [20,21]. Many natural PPARγ ligands have been discovered including AA, prostaglandins, oxidized fatty acid (FA) and some polyunsaturated fatty acid (PUFA) [22–26].

Activation of hepatic PPARγ leads to an upregulation of free FA (FFA) uptake by increasing the expression of fatty acid transporter CD36 [27]. PPARγ is also involved in lipid droplets (LD) formation through increased expression of LD-associated proteins such as perilipin 2 (Plin2) and cell death-inducing DFFA-like effectors (Cide) A and C [28–30]. These LD-associated proteins down-regulate LD lipolysis by reducing association of lipases with the surface of the LD [31–33]. On the other hand, hepatic PPARα regulates energy combustion [34] by activating the mitochondrial and the peroxisomal β-oxidation pathways as well as the microsomal ω-oxidation pathway [35]. Paradoxically, PPARα also activates lipogenesis by regulating the sterol regulatory element binding protein-1 (SREBP-1c) and liver X receptor α expression (LXRα) [36].

Many studies have demonstrated a link between elevated PPARγ expression and hepactic steatosis. Adenoviral over-expression of PPARγ1 in PPARα knockout (KO) mice displaying reduced fatty acid oxidation in liver, induces ectopic fat accumulation and lipogenesis leading to hepatic steatosis [37]. In *Ob/Ob* and lipoatrophic mice, elevated expression of PPARγ2 is associated with non-alcoholic fatty liver disease (NAFLD) while inhibition of PPARγ expression reduces hepatic steatosis through downregulation of lipogenesis and inhibition of LD formation [38–40].

Lipogenesis is regulated at various levels. SREBP-1c and LXRα are the main transcription factors responsible for the induction of acetyl-CoA carboxylase (ACC) and fatty acid synthase (FAS) expression, the two rate-limiting enzymes of lipogenesis. These enzymes produce non-esterified FA (NEFA) that are subsequently desaturated by the stearoyl-CoA desaturase (SCD1). These NEFA are further esterified to form the triglycerides (TG) by enzymes such as the diglycerol acyltransferase (DGAT) [41]. Lipogenesis can be inhibited by AMP-activated protein kinase (AMPK) through phosphorylation and inhibition of both ACC and SREBP-1c [42].

In the present study, we demonstrate that H-apoD Tg mice express significant amounts of H-apoD in the liver. As a result, these mice develop hepatic steatosis through over-expression and activation of PPARγ1. Consequently, the expressions of Plin2, Cide A and C are increased leading to stabilization of LD. In addition, we observed an increase in CD36 expression associated with an elevation of FA uptake. In these conditions, lipogenesis remains unaffected despite an elevated expression of FAS and an inhibition of ACC activity. Overexpressing H-apoD in HepG2 cells in the presence of AA strongly suggests that the presence of hepatic steatosis in Tg mice is the result of PPARγ activation by AA, one of the main ligand of apoD. Supporting this hypothesis, we showed that AA concentration is enriched in liver and decreased in plasma. Our work reveals a novel mechanism of apoD action in lipid metabolism.

## Material and Methods

### Materials

Cell culture medium was purchased from Wisent (Wisent, St-Bruno, Qc, Canada). Bodipy 493/503, Prolong Gold antifade reagent, Galacto-light beta-galactosidase reporter gene assay system, Trizol Reagent and mouse anti-myc monoclonal antibody were purchased from Invitrogen (Invitrogen, Burlington, ON, Canada). AA, anti-mouse horseradish peroxidase-conjugated secondary antibody, luciferin and propidium iodure were obtained from Sigma (Sigma-Aldrich, Oakville, ON, Canada). Anti-PPARγ (C26H12), anti-AMPKα, anti-phospho-AMPKα (Thr172)(40H9), HPRT and β-actin antibodies were from Cell signaling (cell signaling technology, Danvers, MA, USA). Anti-ACC and anti-phospho-ACC (Ser79) antibodies were purchased from Millipore (Millipore, Billerica, MA, USA). Anti-Plin2 antibody was bought from Novus Biological (Novus Biologicals, Littleton, CO, USA) and goat anti-rabbit horseradish peroxidase-conjugated secondary antibody and Bradford reagent were acquired from Bio-rad (Life Science Bio-rad, Mississauga, Ontario, Canada). The antibody against mouse apoD was purchased from Abcam. The H-ApoD monoclonal antibody has already been described [7,43]. Complete Protease Inhibitor Cocktail Tablets were purchased from Roche (Laval, PQ, CAN). Collagenase Type I was acquired from Worthington (Lakewood, NJ). Gal4-PPARγ and UAS-Luciferase plasmids were generously provided by Dr. Maurizio Crestani (University of Milano, Italia).

### Animals

All the experimental procedures were approved by the Animal Care and Use Committee of Université du Québec à Montréal. Animals were housed at 24 ± 1°C in a 12h light dark cycle and fed a standard rodent chow *ad libitum* with free access to water. The H-apoD Tg mice in a C57BL/6 background overexpress the H-ApoD gene under the control of the neuron-specific Thy-1 promoter [13–15]. All experiments were carried out on 12 month old males.

### Preparation of primary hepatocytes

Primary hepatocytes were isolated by *in situ* liver perfusion and collagenase digestion as previously described [44]. Briefly, mice were anaesthetized by intraperitoneal injection of pentobarbital and the portal vein was cannulated. The liver was then perfused with perfusion buffer (10 mM HEPES, 142mM NaCl, 6,7mM KCl; pH 7,85) containing 0,6mM EGTA and 1,5 U/mL heparin and subsequently digested with 30 000U collagenase type I (Worthington) dissolved in 150 mL of perfusion buffer containing 5 mM calcium. Hepatic cells were gently released from the Glisson capsule and incubated for 1h at room temperature with 5X Wash solution consisting of DMEM/F12 (Life technologies, Gibco) with 10% fetal bovine serum (Life technologies, Gibco), 500 U/mL penicillin, 500 μg/mL streptomycin and 1,25 μg/mL Fungizone (Life Technologies) by an orbital shaker. 1x10^6^ cells were seeded on collagen-pretreated plates (Corning Costar) in DMEM/F12 media containing 10% FBS, 100 U/mL penicillin and 100 μg/mL streptomycin. The next day, culture media was removed and renewed with serum-free DMEM/F12 containing the same antibiotics. The cells were starved for 48h prior to the experiments.

### Transfection of HepG2 cells

The human hepatocarcinoma cells (HepG2) were cultured in Eagle’s Minimum Essential Medium (EMEM) supplemented with 10% FBS. Cells were then transfected with a UAS-Luciferase construct (a luciferase reporter plasmid containing five PPAR response elements) in combination with a human myc-tag apoD cDNA construct [43] (or an empty vector) in the presence or not of Gal4-PPARγ (containing the PPARγ cDNA and the DNA binding domain of GAL4) using Fugene HD transfection reagent. 48h after the transfection, cells were incubated for 4h with 7μM of AA bound to BSA at a mole ratio AA:BSA 4:1. Cells were then harvested and cellular extracts were prepared for luciferase [45] and β-galactosidase assays (Invitrogen).

### RNA extraction and semi-quantitative RT-PCR

Tissues were collected, frozen in dry ice and kept at −80°C until further use. Total RNA was extracted with the TRIZOL reagent according to the manufacturer instructions. Total RNA was then reverse transcribed using Transcriptor First Strand cDNA Synthesis Kit and amplified with a Taq DNA polymerase and specific primers (S1 Table). HPRT was used as control.

### Immunoblotting

Tissues or cultured cells were homogenized in cold lysis buffer (50 mM Tris·HCl pH 7.3, 150 mM NaCl, 5 mM EDTA, 0.2% Triton X-100, 2 mM sodium orthovanadate and 10% Complete protease inhibitor). Lysates were then incubated 30 min at 4°C, cleared by centrifugation and stored at –80°C until further use. Based on Bradford assay [46], 50 μg of protein of each sample were separated on SDS-PAGE and transferred onto PVDF membranes. After blocking with 5% milk, 1h at room temperature, the membranes were incubated with the primary antibodies overnight at 4°C. Dilutions of the primary antibodies were: 1:1000 for PPARγ (C26H12), 1:1000 for total AMPKα antibody; 1:1000 for phospho-AMPKα (Thr172) (40H9) antibody; 1:1000 for ACC antibody; 1:300 for anti-phospho-ACC (Ser79) antibody; 1:5000 for Plin2 antibody, 1:10000 for H-apoD antibody [43], 1: 1000 for the mouse apoD (m-apoD) antibody, 1:10000 for HPRT antibody and 1:100000 for β-actin antibody. Primary antibodies were then detected with a goat anti-rabbit horseradish perioxidase-conjugated secondary antibody (1:10000) and visualized by chemiluminescence. Amidoblack staining was used as loading control. Briefly, membranes were stained for 20 min in amidoblack solution (0,1% Amidoblack, 40% v/v methanol and 10% v/v acetic acid) and washed 10 min twice in decoloration solution (40% v/v methanol and 10% v/v acetic acid). Bands were quantified by densitometry using the image J software.

### Indirect ELISA assay

Human apoD was quantified in plasma and liver homogenate of transgenic Tg-apoDH mice by Elisa. 96-well ELISA plates were coated overnight at 4°C with purified human apoD standards (with range concentration of 0–10 μg/mL) and samples (for plasma, 10 μL and for liver, 5 μg) diluted in 0.1M sodium carbonate pH 9.5 to a final volume of 100 μL. The coated wells were blocked with 3% BSA for 1h at RT and were incubated overnight at 4°C with an antibody against biotinylated human apoD (1:10000, biotinylated H-apoD antibody (43)). After washing, the wells were treated with HRP-streptavidin (1:25000) for 1h at RT. After washing, peroxidase substrate TMB (3,3’, 5,5’-Tetramethylbenzidine) solution (100 μL, Fitzgerald, MA, USA) was applied to each well for 30 min and the reaction was stopped by adding 50 μL of 1M phosphoric acid. Absorbance values (at 450 nm) were obtained with Elisa Plate Reader (Tecan Infinite M1000, Tecan US, NC, USA).

To measure the concentration of Prostaglandin E2 (PGE2), whole blood samples were collected from 1 year-old WT and Tg mice by cardiac puncture using heparinized syringe and kept on ice. Plasma was isolated by centrifugation (2000 RPM for 10 minutes at 4°C) and stored at -80°C. Liver extracts were prepared by homogenizing tissues in cold lysis buffer (50 mM Tris-HCl pH 7.3, 150 mM NaCl, 5 mM EDTA, 0.2% Triton X-100, 2 mM sodium orthovanadate and 10% Complete protease inhibitor). Lysates were then incubated 30 min at 4°C, cleared by centrifugation and stored at –80°C. The concentration of PGE2 was then measured using a specific immunoassay (Enzo Life Sciences, ADI-900-001) according to the manufacturer protocol.

### Lipid staining

Liver samples were incubated overnight at 4°C in 4% paraformaldehyde, frozen in dry ice and kept at −80°C until further use. 4 microns thick longitudinal sections were sliced with a cryostat and incubated 5 minutes in a solution of PBS containing 0.04 mg/ml propidium iodide and 0.1 μg/ml Bodipy 493/503. After 3 washes of 5 min in PBS, coverslips were mounted onto slides using Prolong Gold antifade reagent and observed within 24h with a laser scanning confocal microscope (Nikon TE300) (original magnification X60). Lipid droplets were visualized and quantified using image J software.

### In vivo lipogenesis

1-year-old mice fed *ad libitum* were injected intraperitoneally with 7 μCi of ^3^H₂O. One-hour post-injection, animals were sacrificed and the blood and liver were collected. To evaluate the presence of radioactivity, 20 μl of plasma was diluted in 4 ml of Scintillator (Ultima Gold, from Perkin Elmer) and counted with a scintillation counter (TRi Carb 2800TR). To evaluate the fatty acid specific radioactivity, 1g of liver was homogenized in 30% KOH at 70°C. 3 ml of ethanol 96% was added and the samples were heated at 70°C for 2h and acidified with 3 ml of sulfuric acid 9 M. Lipids were extracted 3 times with 10 ml of light petroleum, washed 3 times with 10 ml of water, and dried at RT. Lipids were then mixed in 15 ml of scintillator and counted as described previously. Fatty acid specific radioactivity was expressed as cpm/g of liver and counted. The rate of lipogenesis was calculated by dividing the fatty acid specific radioactivity by the plasma water specific activity.

### ^3^H-oleate fatty acid uptake

Primary hepatocytes from WT and Tg mice were cultured in serum free media for 48h. Fatty acid uptake was measured using ^3^H-oleate as previously described [47]. Briefly, cells were incubated in 0.68 μCi/mL ^3^H-oleate (50 μM)_bound to BSA (fatty acid/BSA molar ratio 2:1) in serum free DMEM/F12 media for 10 min at RT. The reaction was stopped by adding 200 μM of ice-cold phloretin solution for 2 min. Cells were then washed 3 times with PBS and lysed in 0.1 N NaOH for 30 min at RT. Radioactivity in lysates was counted in 10 mL Ultima-Gold solution (Tri-Carb 2800TR, Perkin Elmer) and protein were quantified (Bradford Assay, BioRAD).

### Fatty acid (FA) profiling

FA composition was measured by a modified gas chromatography-mass spectrometry (GC-MS) method, as previously described [48]. Briefly, total lipids were extracted from plasma with a mixture of methyl-tert-butyl ether (MTBE), methanol and water [49]. For liver, pulverized tissues (25mg) were incubated overnight at 4°C in a solution of chloroform/methanol (2:1) containing 0.004% butylated hydroxytoluene (BHT), filtered through gauze and dried under nitrogen gas. Plasma and liver FA were analyzed as their fatty acid methyl derivatives (FAME) after direct transesterification with acetyl chloride/methanol [50]. Injections (2 μL for plasma and 1 μL for liver samples) were performed onto an Agilent 7890B gas chromatograph equipped with a Select FAME CP7420 capillary column (100 m; 250 μm inner diameter; 230 μm thickness) coupled with a 5977A Mass Selective Detector operated in positive chemical ionisation mode using ammonia as reagent gas. FA were identified according to their retention time and *m/z*, and their concentration was calculated using a mix of internal and external labelled standards added to liver and plasma samples at known concentrations. The concentration of arachidonic acid, calculated using its [^2^H_8_]-labeled counterpart as internal standard, is reported as absolute concentration (μM or nmol/mg tissue) and relative to total fatty acid content (%).

### Statistical analysis

Results are expressed as means ± SD. Statistical analysis was performed with GraphPad 5 software. The statistical significance from control values was determined by Student's t-test. Values were considered to be significant at P ≤0.05.

## Results

### Elevated PPARγ and C/EBP expression in liver of H-apoD Tg mice

In the present study, we used a H-apoD Tg mice where the transgene is driven by the neuron specific Thy-1 promoter (14). Despite a predominant expression in the central nervous system, a significant mRNA expression was detected in both plasma and liver (for liver, 20% of the level detected in the hippocampus). The H-apoD protein is also detected in the plasma (WT mice were used as a negative control) (0.5 mg /100 ml of plasma) and in liver (0.7 ng/mg protein) of Tg mice. We also detected the endogenous protein in the blood but not in the liver (S1 Fig). Therefore, the hepatic H-apoD protein can originate either from an endogenous hepatic expression or from a selective blood uptake.

**Fig 1:**
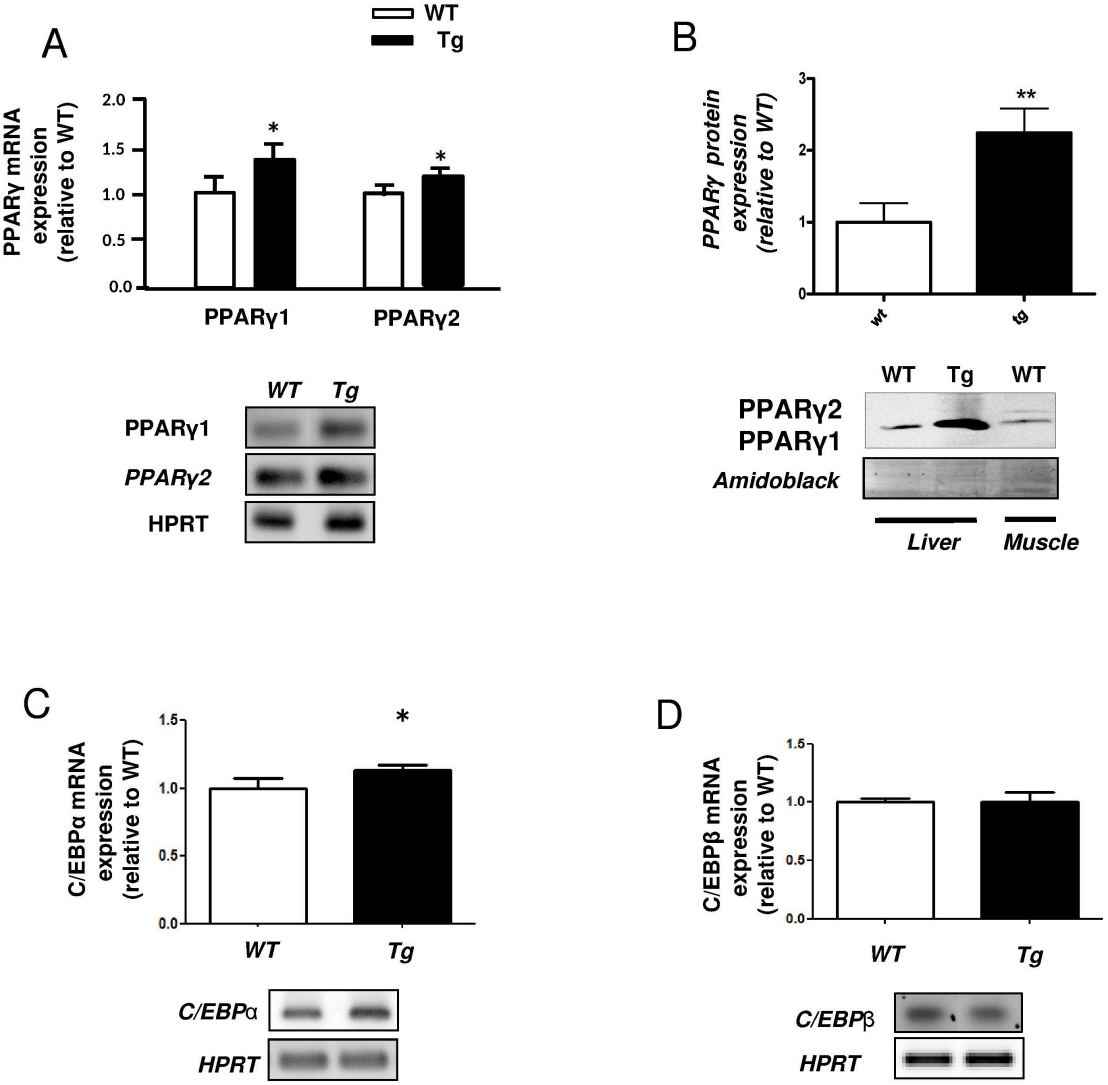
PPARγ and C/EBP expression in the liver of H-apoD Tg mice. Semi- quantitative RT-PCR (**A**) and Western blot (**B**) analysis of PPARγ expression in liver and skeletal muscle of WT and H-apoD Tg mice. **A**- Graphs represent the mRNA expression level normalized by HPRT. A representative gel is presented above. **B-** The graph represents the level of PPARγ protein expression standardized by amidoblack staining. Muscle tissue was used for PPARγ1/PPARγ2 positive control. Semi-quantitative RT-PCR analysis of C/EBPα (**C**) and C/EBPβ (**D**) mRNA expression. The graphs represent the level of mRNA expressions normalized by HPRT. Values are expressed relatively to the WT mice and are the means ± SD of 4 mice per group. *P<0.05 and **P<0.01 vs WT mice.

Using the H-apoD Tg mice, we previously demonstrated that hepatic PPARγ mRNA was increased [15]. In the present study, we showed that both PPARγ1 and γ2 mRNA levels are increased (1.37-fold and 1.16-fold Tg *vs* WT respectively) (Fig 1A). At the protein level, PPARγ1 was increased by 2.24-fold in Tg mice while PPARγ2 was poorly detected (Fig 1B). The expression of C/EBPα mRNA, an early marker of adipogenic-like phenotype and a PPARγ target gene was also increased (Fig 1C) while C/EBPβ, which is not a PPARγ target, remained unchanged (Fig 1D).

### Lipid droplets formation

**Fig 2:**
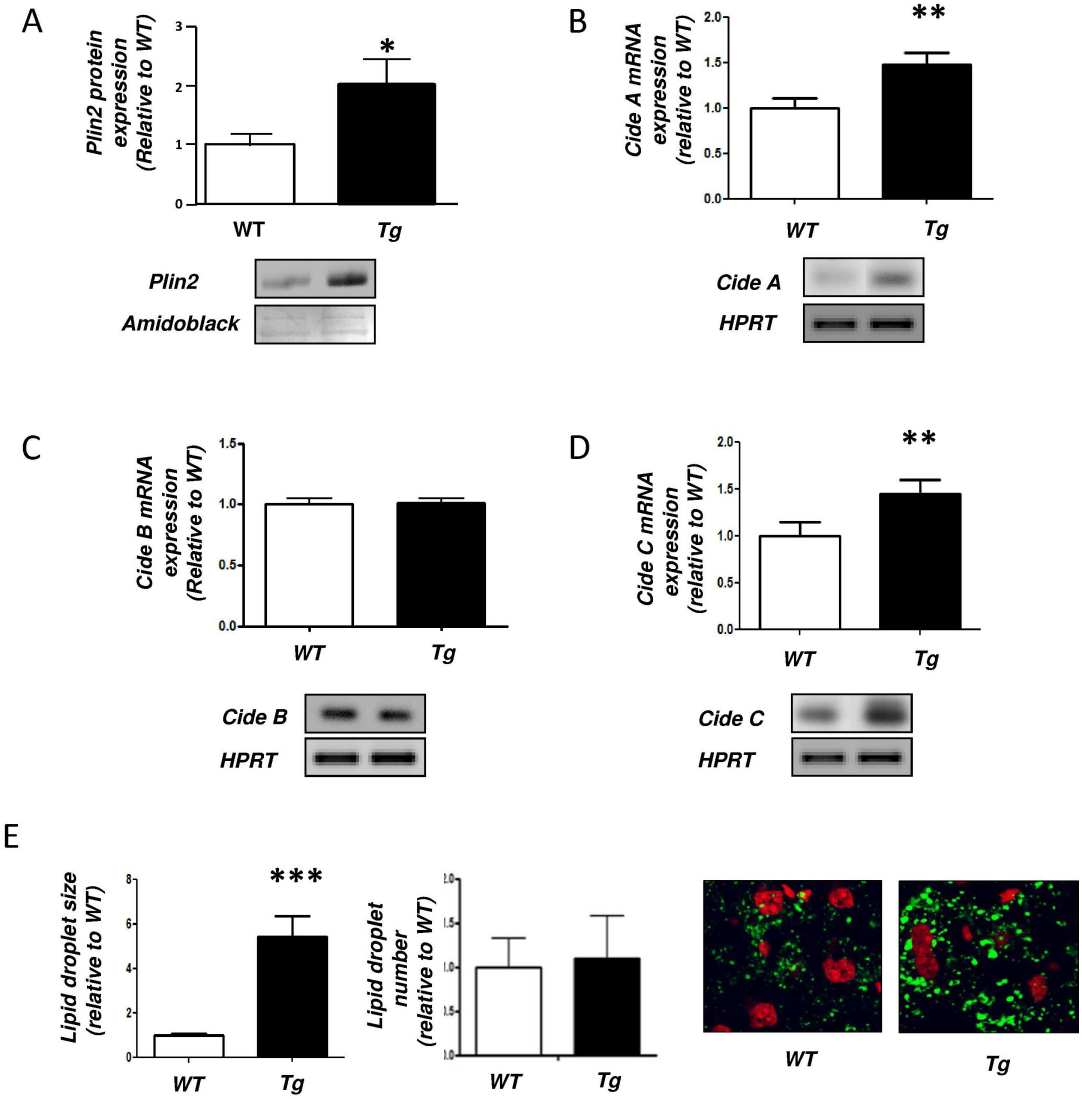
Lipid droplets formation in the liver of H-apoD Tg mice. **A**- Western blot analysis of Plin2 expression. The graph represents the level of Plin2 protein expression standardized by amidoblack staining. A representative gel is presented. Semi-quantitative RT-PCR analysis of Cide A (**B**), Cide B (**C**) and Cide C (**D**) mRNA expression. The graphs represent the level of mRNA expressions normalized by HPRT. Values are expressed relatively to the WT mice and are the means ± SD of 4 mice per group. **E**-Confocal analysis of lipid droplets in liver tissues of WT and H-apoD Tg mice. Lipid droplets are stained with bodipy (in green) and nucleus with propidium iodide (in red). Graphs represent the quantification of 18 images. *P<0.05, **P<0.01, P<0.001 vs WT mice.

We then measured the expression level of key proteins known to be involved in LD formation. The expression of the PPARγ target gene Plin2 [51] was increased by 1.98-fold in Tg mice (Fig 2A). Similar observations were made regarding Cide A and Cide C (1.47 and 1.45-fold respectively), two other targets of PPARγ that are implicated in LD fusion [52] (Fig 2B and 2D). A well-documented independent gene of PPARγ regulation, Cide B remained unchanged (Fig 2C). Conversely, the expression of genes coding for several lipases (adipose triglyceride lipase (ATGL), hormone sensitive lipase (HSL) and monoglyceride lipase (MGL)) as well as for the ATGL coactivator comparative gene identification 58 (CGI-58) remained identical (*data not shown*). Consistently with an elevated expression of proteins involved in hepatic LD formation and fusion, we found that the size of the hepatic LD in H-apoD Tg mice was drastically increased (5.45-fold) compared to WT. However, we did not detect a significant modification in the number of LD between the Tg and WT mice suggesting that the observed phenomenon was a result of both LD fusion and formation of new LD (Fig 2E).

### Hepatic FFA uptake

**Fig 3:**
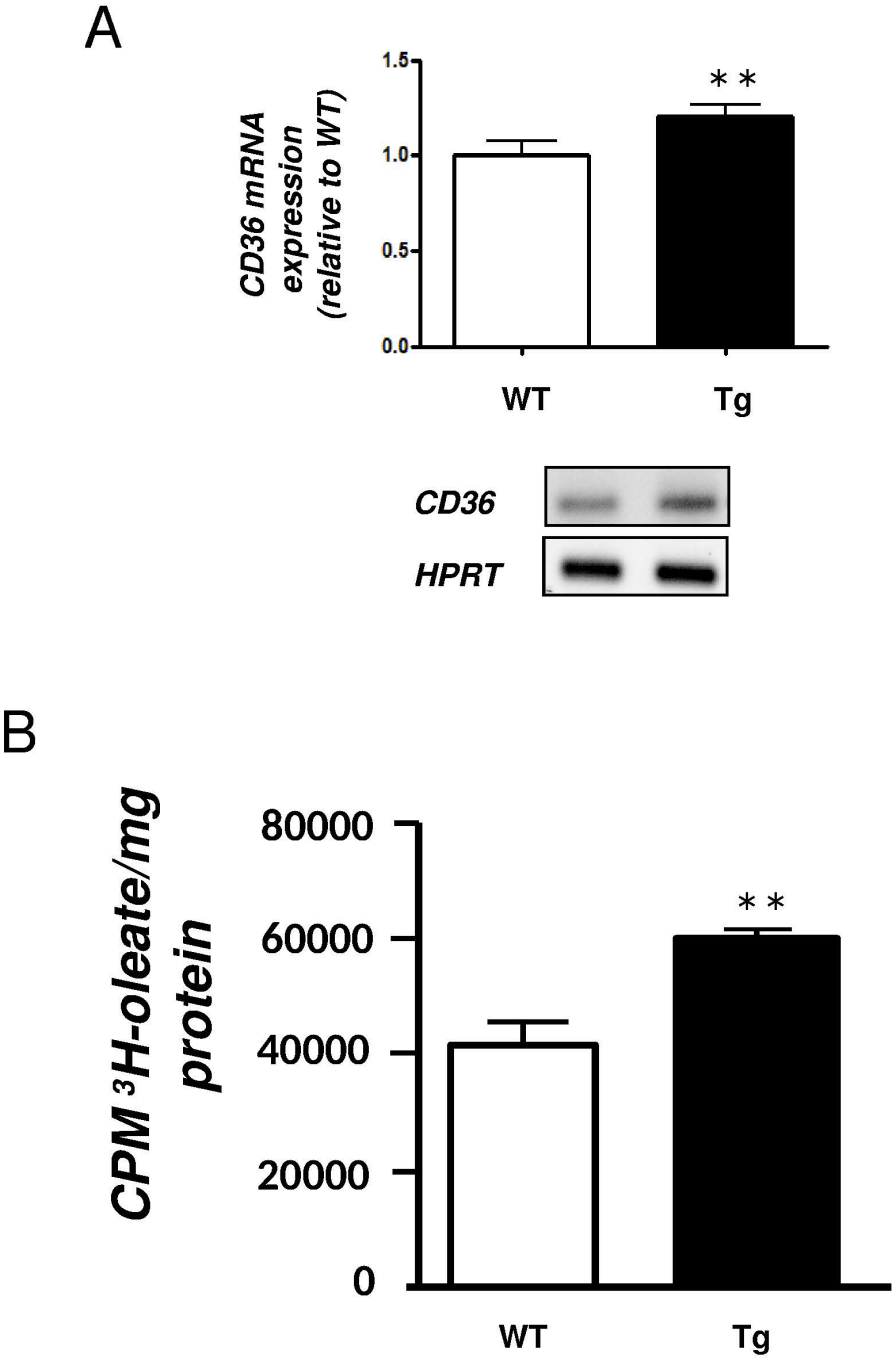
FFA uptake in the liver of H-apoD Tg mice. **A-** Semi-quantitative RT-PCR analysis of CD36 expression in liver tissue from WT and H-apoD Tg mice. Graph represents the mRNA expression levels normalized by HPRT. Representative gels are presented. Values are expressed relatively to the WT mice and are the means ± SD of 4 mice per group. **B**- ^3^H-oleate uptake was evaluated in primary hepatocytes prepared from WT and Tg mice. Results are expressed as CPM of ^3^H per mg of hepatic protein and represent the mean of 3 independent experiments. **P<0.01 *vs* WT mice.

We previously demonstrated that circulating FFA, cholesterol and TG concentrations were not different between H-apoD Tg and WT mice [15]. The mRNA expression of two enzymes implicated in lipoprotein metabolism, lipoprotein lipase (LPL) and hepatic lipase (HL) remained also unaffected in Tg mice (*Data not shown*). In contrast, the expression of CD36, a target of PPARγ and the main transporter of hepatic FFA in cells, was significantly increased (1.2-fold) in the liver of Tg mice (Fig 3A). To evaluate the effect of elevated CD36 expression on hepatic FA uptake in Tg mice, we prepared primary hepatocytes from both WT and Tg animals. Incubation of cells with ^3^H-oleate showed a 30% increase in oleate uptake in Tg mice (Fig 3B). This confirmed that the upregulation of CD36 provides a functional role in those mice.

### Hepatic lipogenesis

**Fig 4:**
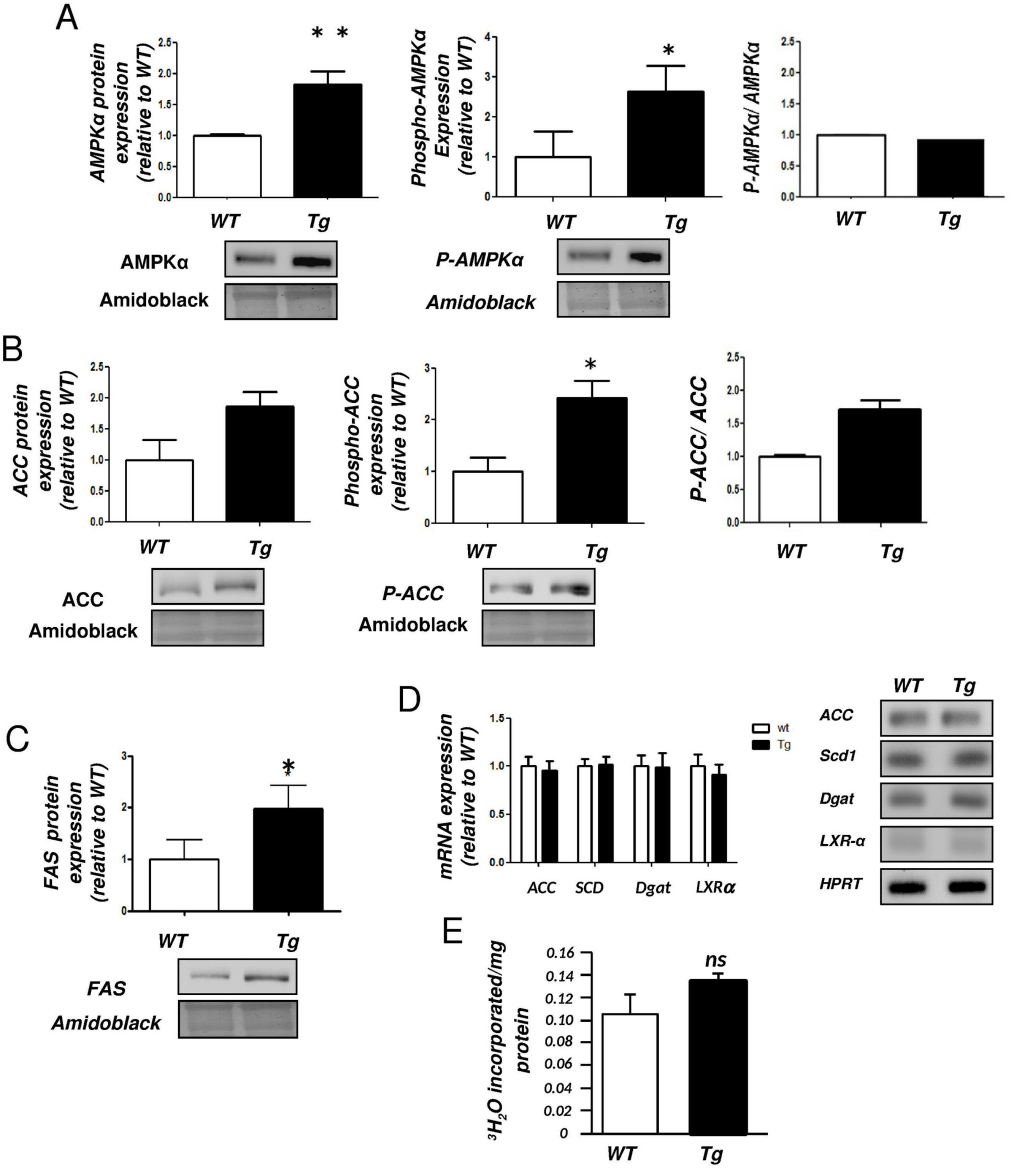
Lipogenesis in the liver of H-apoD Tg mice. Western blot analysis of total and phospho-AMPKα (**A**), total and phospho-ACC (**B**) and FAS (**C**) protein expression in the liver of WT and H-apoD Tg mice. The graphs represent the levels of protein expressions standardized by amidoblack staining. Representative gels are presented. **D-** Semi-quantitative RT-PCR analysis of ACC, SCD1, DGAT and LXRα mRNA expression. The graph represents the level of mRNA normalized by HPRT. Representative gels are presented. **E**- *In vivo* lipogenesis measured in 1 year old mice. The values represent the amount of ^3^H_2_O incorporated into triglycerides. Values are expressed relatively to the WT mice and are the means ± SD of 4 mice per group. *P<0.05, **P<0.01 vs WT mice.

We previously demonstrated that the mRNA levels of SREBP-1c and FAS were increased in the liver of H-apoD Tg compared to WT mice [15]. Since elevated lipogenesis has also been associated with hepatic steatosis, we evaluated the expression levels and the activity of several other key proteins involved in hepatic lipogenesis. We showed that the AMPK expression was increased by 1.82-fold in the liver of Tg mice. Its phosphorylation on Thr172 residue was also increased (2.63 fold), but the AMPK activity was unaffected as evaluated by the ratio of P-AMPα/AMPKα (Fig 4A). As a consequence of increased AMPKα phosphorylation in Tg mice, one of the AMPKα target proteins, ACC displayed higher phosphorylation levels on Ser79 (1.7 fold, measured by the ratio P-ACC/ACC) suggesting a reduced activity of the first lipogenic enzyme (Fig 4B). On the other hand, a significant increase in FAS protein expression was observed (1.97-fold) complementing our previous observation at the mRNA level [15] (Fig 4C). However, the mRNA expression of ACC, SCD1, DGAT and LXRα remained unaffected in the liver of H-apoD Tg mice compared to WT (Fig 4D).

As an increased phosphorylation of ACC and an augmented expression of FAS seemed contradictory, we measured the *de novo* lipogenesis *in vivo* by ³H_2_O injection in mice. As shown in Fig 4E, despite a trend, the level of *de novo* lipid synthesis in the liver is not significantly different between Tg and WT mice suggesting that hepatic steatosis cannot be attributed to a significant modification of *de novo* lipid synthesis.

### Hepatic β-oxidation

**Fig 5:**
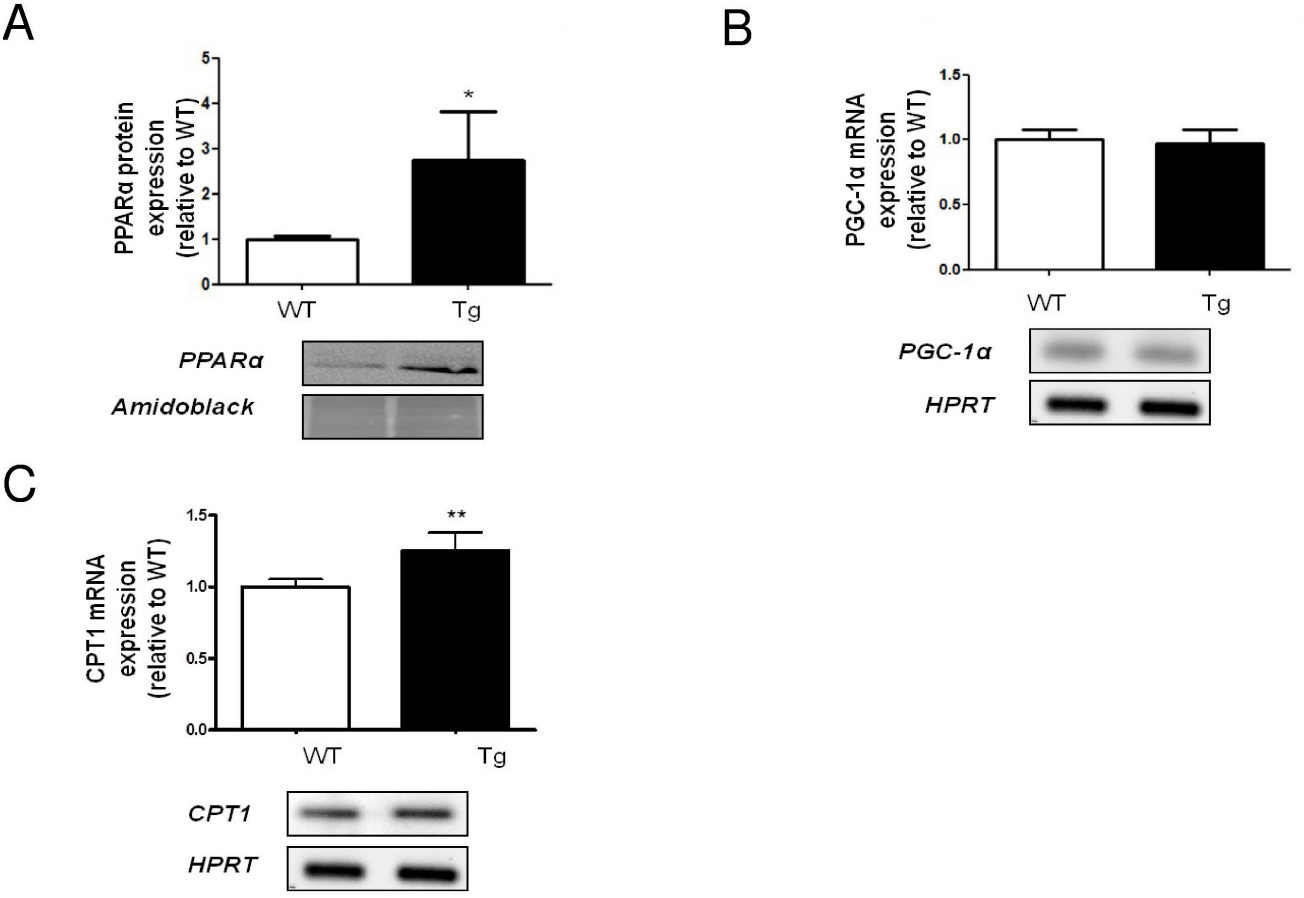
Analysis of genes involved in β-oxidation in the liver of H-apoD Tg mice. **A**- Western blot analysis of PPARα protein expression. The graph represents the level of PPARα protein expression standardized by amidoblack staining. A representative gel is presented. Semi-quantitative RT-PCR analysis of PGC-1α (**B**) and CPT1 (**C**) expression in liver of WT and H-apoD Tg mice. PGC1α and CPT1 gene expression was normalized by HPRT. For each graph, the H-apoD Tg values were normalized by the WT values and are the means ± SD of 4 mice per group. *P<0.05 and **P<0.01 *vs* WT mice.

We previously demonstrated that PPARα mRNA was increased in H-apoD Tg mice liver suggesting an elevated hepatic lipid β-oxidation [15]. A similar increase was observed at the protein level (2.73 fold) (Fig 5A). Since PPARα is known to regulate the expression of genes involved in the β-oxidation pathway, we examined the expression of two key proteins involved in this process. The mRNA of PGC-1α, a co-activator of PPARα remained unchanged (Fig 5B). However, the mRNA expression of the carnitine palmitoyltransferase I (CPT-1), the rate limiting enzyme of the mitochondrial lipid transfer was slightly increased (1.26-fold) in the liver of H-apoD Tg mice compared to WT (Fig 5C). This might be associated to a slight upregulation of lipid β-oxidation in the liver of H-apoD Tg mice.

### Effect of apoD overexpression on PPARγ activation by AA

**Fig 6:**
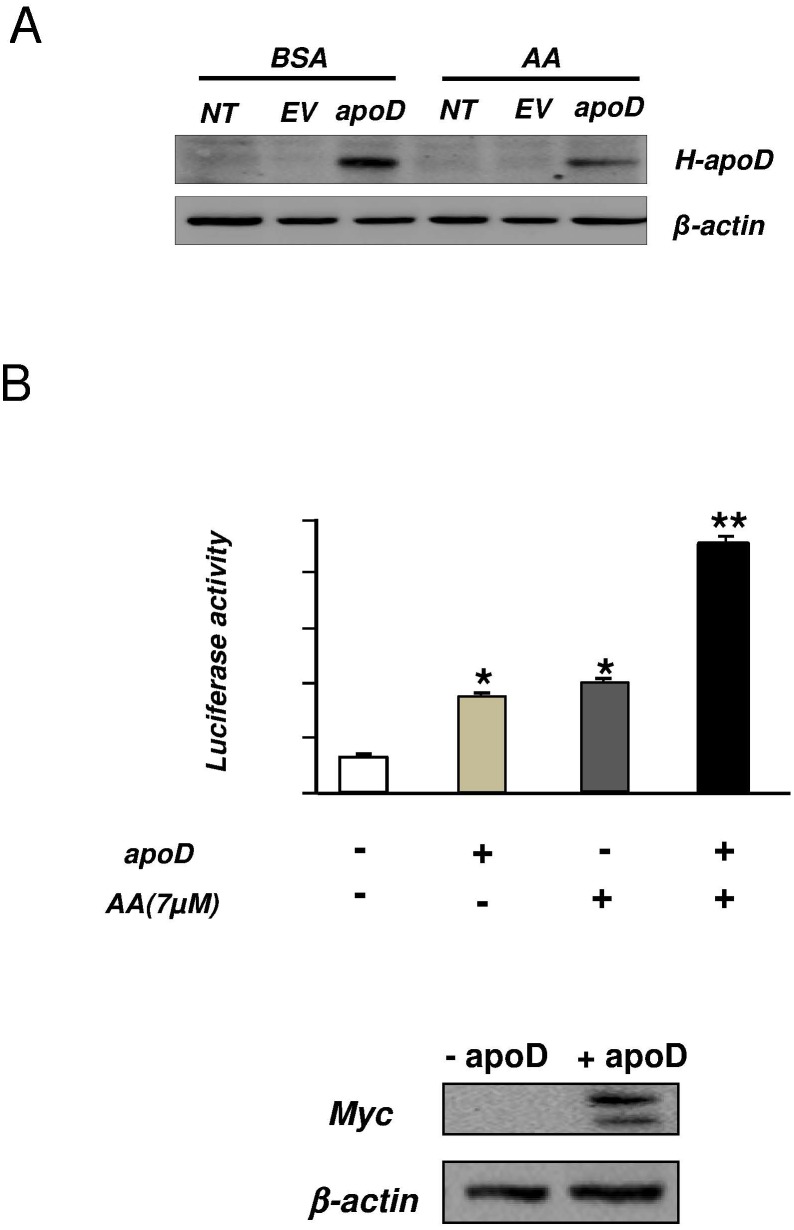
PPARγ transcriptional activity in presence of AA and/or apoD. **A-** HepG2 cells were either non transfected (NT) or transfected with a myc-Tag apoD-cDNA or empty vector (EV) construct and incubated with BSA or arachidonic acid (AA). The level of H-apoD expression was evaluated by Western blot using a specific H-apoD antibody. **B**- HepG2 cells were transfected with UAS-Luc, GAL4-PPARγ, β-galactosidase and with either an empty vector or a myc-Tag apoD-cDNA construct. After transfection, cells were treated with 7 μM AA for 4h. Luciferase activity represents data normalized by β-galactosidase activity. The data represent the mean ± SD (n = 3). *P<0.05 and **P<0.01 *vs* the non-stimulated control without apoD. The gel presented below showed the expression of apoD in transfected cells using a myc antibody.

To understand the link between apoD and PPARγ over-expression and activation, we evaluated the potential role of apoD as an AA transporter, one of the main ligands of PPARγ. We used the human hepatocarcinoma HepG2 cell line as a well-characterized model for the study of hepatic lipid metabolism. Cells were transfected with a construct containing the cDNA of human apoD. No apoD was detected in cells either untransfected or transfected with an empty vector. Transfection with the H-apoD cDNA showed a strong expression of the protein (Fig 6A). We next co-transfected the H-apoD cDNA construct with a construct containing five peroxisomes proliferator-activator receptor elements (PPRE) linked to a luciferase reporter gene. Thereafter, cells were incubated with 7 μM of AA in presence or absence of apoD. At this concentration, AA does not induce any cellular toxicity [53] but a slight decrease in apoD expression (Fig 6A). Over-expression of apoD increased PPARγ transcriptional activity (3.7 fold, Fig 6B). Addition of AA alone increased PPARγ transcriptional activity to a similar extent (3.9-fold). Very interestingly, a combination of AA and apoD over-expression showed a very strong synergistic transactivation effect on PPARγ transcriptional activity (approximately 9-fold) (Fig 6B).

### Consequence of apoD overexpression on plasma and hepatic AA concentration

**Fig 7:**
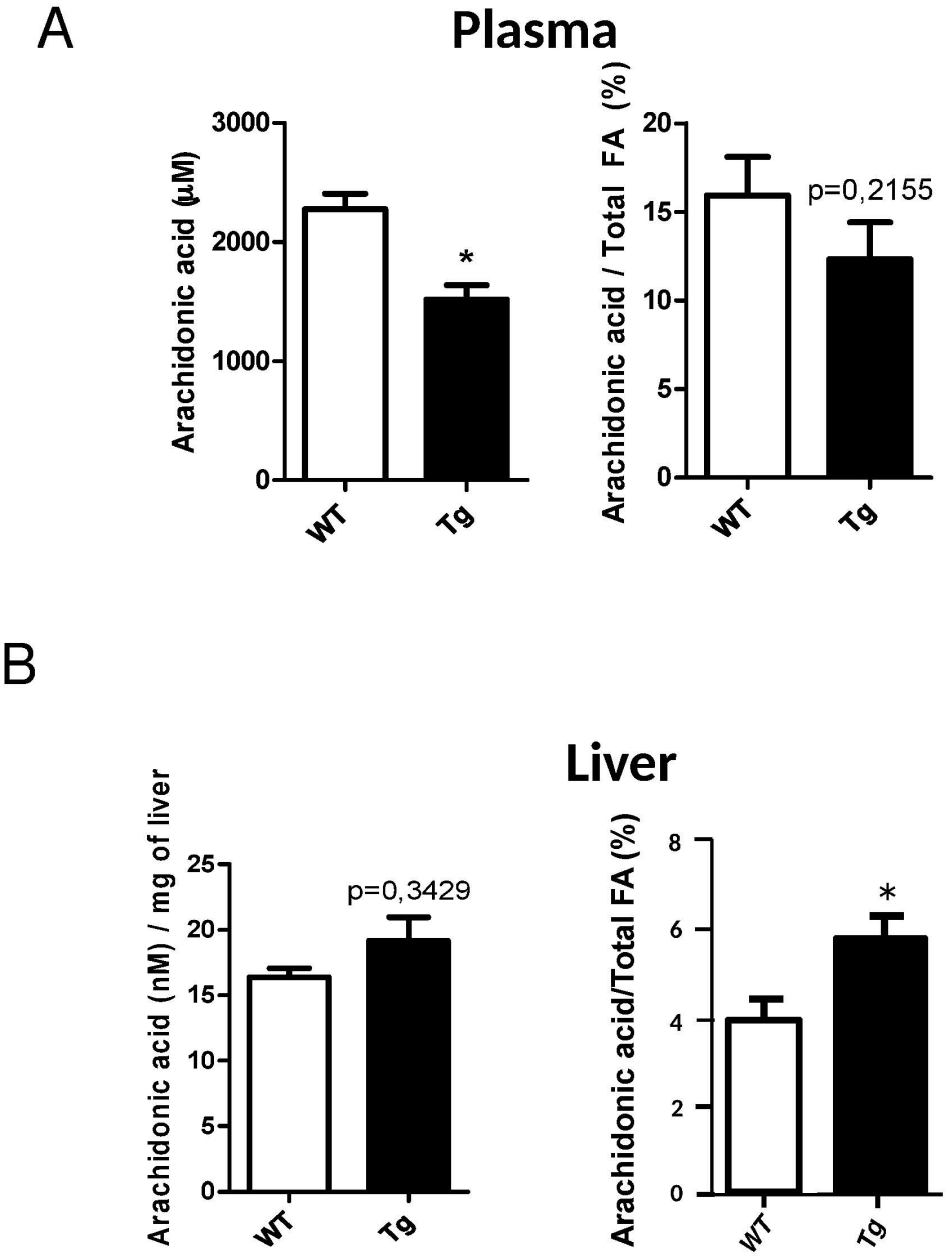
Concentration of plasmatic and hepatic AA in WT and H-apoD mice. The concentration of arachidonic acid was evaluated by isotope dilution gas chromatography-mass spectrometry and reported in A) plasma as absolute values (left panel: μM) and relative to total fatty acid content (right panel: %); and in B) liver as absolute values (left panel: nmol/mg tissue wet weight) and relative to total fatty acid content (%). The data represent the mean ± SD (n = 3 for plasma and 3 for liver). *P<0.05 *vs* WT mice.

To support our hypothesis on the role of apoD in AA transport and the consequence on PPARγ activation, we evaluated the total concentration of AA (free and bound to TG and PL) in plasma and liver of WT and H-apoD Tg mice using quantitative isotope dilution gas chromatography-mass spectrometry. Our data show that the absolute AA concentration is significantly decreased in the plasma of H-apoD Tg compared to WT mice. Consequently, we also observed a significant enrichment in hepatic AA in the total fatty acid pool (Fig 7). However, the prostaglandin E2 concentration is similar between WT and H-apoD Tg mice (S2 Fig)

Taken together, our study shows that overexpression of H-apoD leads to increased hepatic PPARγ expression and subsequent activation of the proteins involved in LD formation. This effect is also associated with an elevation of fatty acid uptake while lipogenesis remains unaffected. Because the metabolic syndrome is mild, the slight increase in the mitochondrial β-oxidation is probably a compensatory mechanism. Experiments performed with HepG2 cells suggest that the hepatic steatosis is a result of an increased AA by apoD in the liver as confirmed by the enrichment of hepatic AA in H-apoD mice. This leads to PPARγ transcriptional activation and downstream effects such as the mild metabolic syndrome and the associated insulin resistance.

## Discussion

The goal of this study was to characterize the molecular mechanisms leading to TG accumulation in the liver of adult H-apoD Tg mice. In mice, apoD is mainly expressed in the CNS while in human, it is expressed in several organs although at different levels [1,12]. As expected, analysis of the expression pattern of human apoD mRNA in Tg mice clearly showed a higher expression in the CNS but expression in the liver and in the plasma was also observed [15]. Similar observations were made at protein levels. The hepatic steatosis can therefore be the result of increased apoD concentration in the liver or from the uptake of circulating apoD. Indeed, as previously suggested by the study of Suresh and collaborators using a mice model of the Niemann-Pick type C (NPC) disease, apoD is a circulating protein [54]. Further experiments are needed in order to clearly determine the origin of the hepatic H-apoD protein in our Tg mice.

It was previously shown that apoD interacts with the leptin receptor regulating the hypothalamic function in energy control [55]. It is unlikely that our observations are a result of an overexpression of apoD in the hypothalamus. In the study mentioned here above, the authors observed a strong correlation between the hypothalamic level of apoD, the body fat mass and the circulating level of leptin which was associated with increased food intake. However, in H-apoD Tg mice, we did not observe any changes in body fat mass and in circulating leptin concentration [15]. In addition, the food intake was not modified (*data not shown*). Taken together, this argues for a direct effect of apoD in the liver rather than a role in hypothalamus.

It is also unlikely that our observations are due to an insertion of the apoD transgene in a genomic area disrupting the metabolism, since we observed a similar phenotype in Tg mice where H-apoD expression was driven by the neuron-specific enolase promoter (NSE) [15]. The behavioral, molecular, biochemical and general health characterization demonstrated similar phenotypes between the NSE and the Thy-1 strains. However, we only studied the Thy-1 mice because the hepatic steatosis was more pronounced in this line.

In the present study, we showed that increased expression of H-apoD in the liver activates the nuclear receptor PPARγ, leading to hepatic fat accumulation at one year of age. The development of hepatic steatosis is probably not a consequence of age-dependent insulin resistance as the increased hepatic PPARγ expression is observed as early as 3 months of age in Tg animals (*data not shown*). Despite an increase in hepatic FA uptake in Tg mice, no differences were detected in the plasmatic levels of FA and TG. As such, our hypothesis is that the increase in FA uptake is probably a slow process that does not allow the detection of differences in the plasmatic levels of FA and TG.

Our *in vitro* studies strongly suggest that apoD acts as an AA transporter, leading to the activation of PPARγ. AA is the preferential ligand of apoD [3] and a precursor for prostaglandins which are also natural PPARγ activators [22,23]. We showed that activation of PPARγ by AA in HepG2 cells is significantly potentiated by apoD. In agreement with our study, Thomas *et al*. [56] demonstrated in cultured embryonic kidney (HEK) 293T cells that apoD stabilizes AA at the plasma membrane and inhibits the release of AA in the extracellular media. Here, we show that plasmatic AA is decreased in H-apoD Tg compared to WT mice while hepatic concentration is enriched. Interestingly, increased hepatic concentration of AA has been associated with fatty liver [57] as observed in H-apoD Tg mice.

Challenging our observations, Perdomo *et al*.[58] showed that, in mice injected with an adenovirus expressing apoD, the activation of LPL leads to a decrease in circulating TG-rich lipoproteins. The authors did not observe any accumulation of ectopic fat in the liver. The difference between their study and this one could be attributed to the fact that the adenovirus half-life in mice is certainly too short to allow development of steatosis. Also, the use of adenoviruses to overexpress apoD may lead to a different level of apoD expression in the liver.

In the liver of H-apoD Tg mice, we observed a strong increase in PPARγ expression associated with an activation of its transcriptional activity. Interestingly, PPARγ and C/EBPα activate each other’s expression maintaining a positive feedback loop for the development of an adipocyte like phenotype [20,21,59]. Complementing these data, we observed a slight increase in C/EBPα expression while C/EBPβ remained unchanged. It is to note that the upregulation of C/EBPα expression is minor. This could be explained by the fact that the hepatic steatosis progression in Tg mice is very slow and hence, the genes implicated are expected to be only slightly modulated. Furthermore, elevated PPARγ expression results in the increase of CD36 expression. However, the LPL and HSL levels remained constant at least at the mRNA level. Previous studies demonstrated that activation of PPARγ in the liver increases expression of LPL and CD36 [29,30] however, in our model only the FA uptake is affected without any increase in lipoproteins hydrolysis. Similar observations associating increased CD36 expression and FA uptake were made in cardiac cells [60].

Another mechanism by which PPARγ may be implicated in hepatic lipid accumulation could be by the induction of LD formation and maturation [28–30]. In the present study, we demonstrated that the expression of Plin2, Cide A and C, three targets of PPARγ, was increased in H-apoD Tg mice. At the opposite, the expression of Cide B, which is not a PPARγ target, was unaltered. Listenburger *et al* [33] showed that Plin2 lowers the rate of TG turnover in LD by reducing the association of ATGL with LD and therefore the hydrolysis of TG [61] while Cide A and C are implicated in the fusion of LD [62–65]. This may explain the 5-fold increase in LDs’ size observed in the liver of H-apoD Tg mice.

Several studies showed that activation of PPARγ induces lipogenesis [38–40]. Since we previously showed that SREBP-1c and FAS mRNA expressions were increased in H-apoD Tg mice liver [15], we measured the mRNA levels of key lipogenic enzymes including LXRα, a transcription factor that induces lipogenic gene transcription [66–70]. We did not observe any change in the mRNA levels of ACC, SCD1, DGAT and LXRα. We also observed an elevation of AMPK expression. The increased expression of AMPKα is consistent with a recent study reporting that CD36 increases AMPK expression through the action of both PPARγ and PGC-1α [71]. Consequently, AMPKα phosphorylation is higher in the liver of Tg mice, resulting in increased phosphorylation and inhibition of ACC [72]. Interestingly, Mao *et al* [73] showed that inhibition of ACC1 in mouse liver induces expression of FAS explaining why FAS expression is increased in our conditions. However, by directly measuring *de novo* lipogenesis *in vivo* using ^3^H_2_O, we showed the over-expression of H-apoD has no significant effect on *de novo* lipid synthesis in 1-year-old animals. A similar observation was made in 3-month-old mice (*data not shown*).

PPARα is activated by long chain fatty acid (LCFA) [74,75]. We previously demonstrated that hepatic PPARα mRNA is increased in H-apoD Tg mice liver [15]. PPARα is a nuclear receptor that activates the transcription of several genes implicated in the mitochondrial β-oxidation of lipids [75]. Its elevated expression is associated with an increased expression of CPT1, the rate limiting-enzyme of the mitochondrial β-oxidation [76]. Since CPT-1 is normally inhibited by malonyl-CoA produced by ACC [77], inhibition of ACC in the liver of H-apoD Tg mice is associated with an increased expression of CPT-1 strongly suggesting an activation of the β-oxidation. However, this increased expression is mild and does not appear sufficient to reverse the progression of the hepatic steatosis in the H-apoD Tg mice.

## Conclusion

Our study describes for the first time a role for apoD in the regulation of PPARγ and the downstream activation of metabolic pathways leading to hepatic steatosis. In Tg mice, elevated apoD expression leads to higher hepatic AA concentration and subsequent activation of the nuclear receptor PPARγ. As a result, PPARγ target genes such as CD36, Plin2, Cide A and Cide C are increased leading to an enhanced LCFA uptake by the hepatocytes and protecting LD against lipolysis by blocking access to lipases. Both PPARγ activation and high CD36 expression induce AMPK expression which leads to increased PPARα expression and its downstream target gene, CPT1 which in turn activates mitochondrial β-oxidation. However, the activation of this compensatory pathway is insufficient to fully inhibit the accumulation of ectopic fat in the liver, but it probably contributes to reduce the progression of hepatic steatosis. Overall, our study highlights a new role for apoD as an AA transporter regulating lipid accumulation in the liver.

## Supporting Information

S1 Fig ApoD expression in tissues of WT and Tg mice. The expression of H-apoD mRNA was evaluated by Northern blot analysis in various tissues of H-apoD Tg mice. HPRT was used as a reference. Results are expressed as a percentage of the expression level measured in the hippocampus and standardized by the level of HPRT expression (**Fig. A**). Human apoD was quantified in plasma and liver homogenate of transgenic Tg-apoDH mice by indirect Elisa. Results are expressed as average ± SEM. (**Fig. B**). Western blot analyses of plasma (P) and liver (L) in WT and Tg mice. A polyclonal mouse antibody was used to detect the endogenous apoD protein (**Fig. C**).
(TIF)

S2 Fig Prostaglandin E2 expression in tissues of WT and Tg mice. PGE_2_ levels were measured by Elisa as described in the Material and methods section, in the plasma and the liver of 1 year-old WT and Tg mice. The data are the means ± SEM of 3 mice per genotype.
(TIF)

S1 Table Primers used in semi-quantitative RT-PCR. The table indicates the sequence of the primers used in semi-quantitative RT-PCR analysis presented in the manuscript.
(TIF)
