# Supplementary figures and images for: Apolipoprotein D Transgenic Mice Develop Hepatic Steatosis through Activation of PPARγ and Fatty Acid Uptake
Source: PLoS One. 2015 Jun 17;10(6):e0130230. doi: 10.1371/journal.pone.0130230 (PMC4470830; doi:10.1371/journal.pone.0130230)

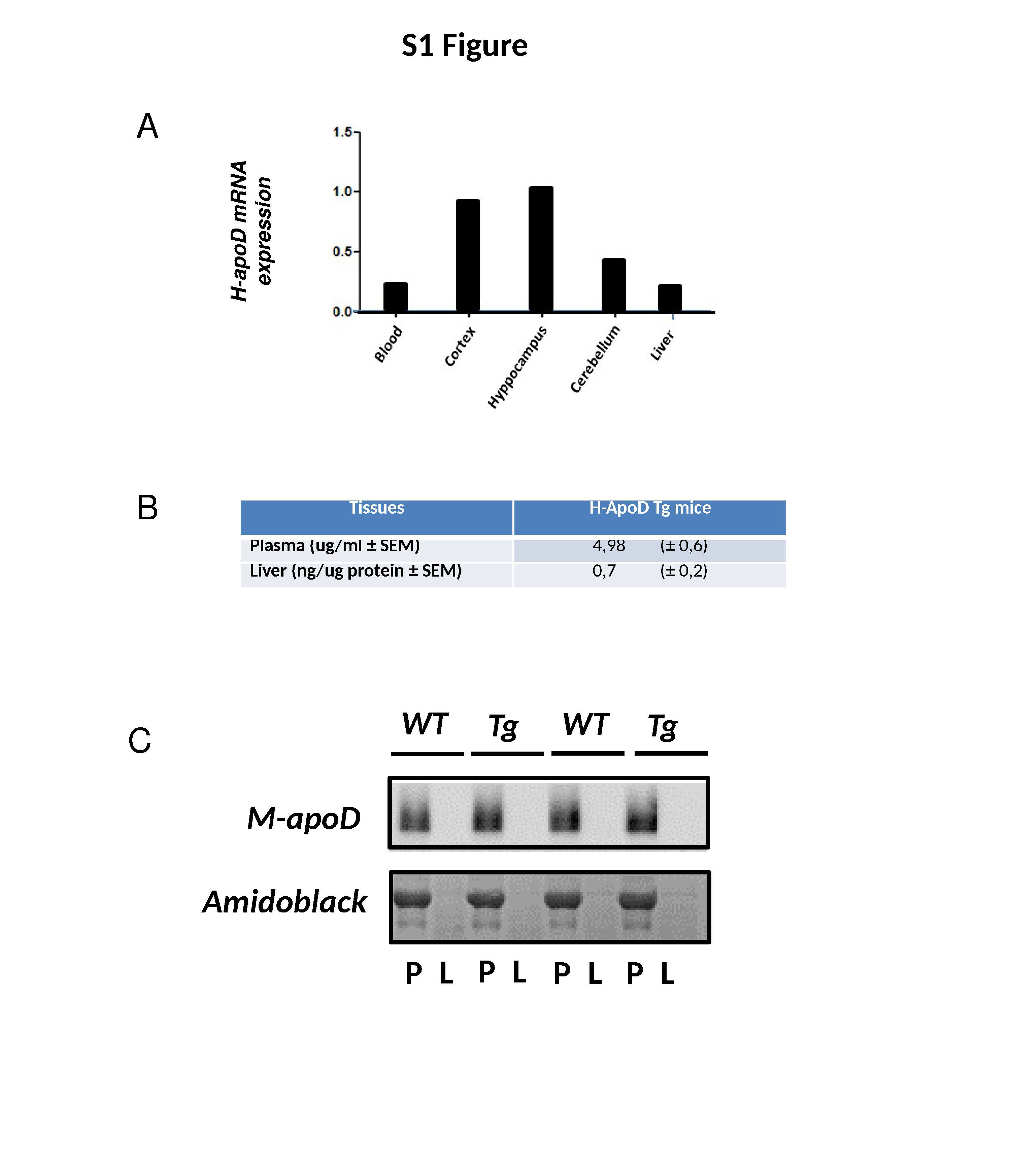

Supplement: S1 Fig — The expression of H-apoD mRNA was evaluated by Northern blot analysis in various tissues of H-apoD Tg mice. HPRT was used as a reference. Results are expressed as a percentage of the expression level measured in the hippocampus and standardized by the level of HPRT expression (Fig. A). Human apoD was quantified in plasma and liver homogenate of transgenic Tg-apoDH mice by indirect Elisa. Results are expressed as average ± SEM. (Fig. B). Western blot analyses of plasma (P) and liver (L) in WT and Tg mice. A polyclonal mouse antibody was used to detect the endogenous apoD protein (Fig. C). (TIF) [file pone.0130230.s001.tif]

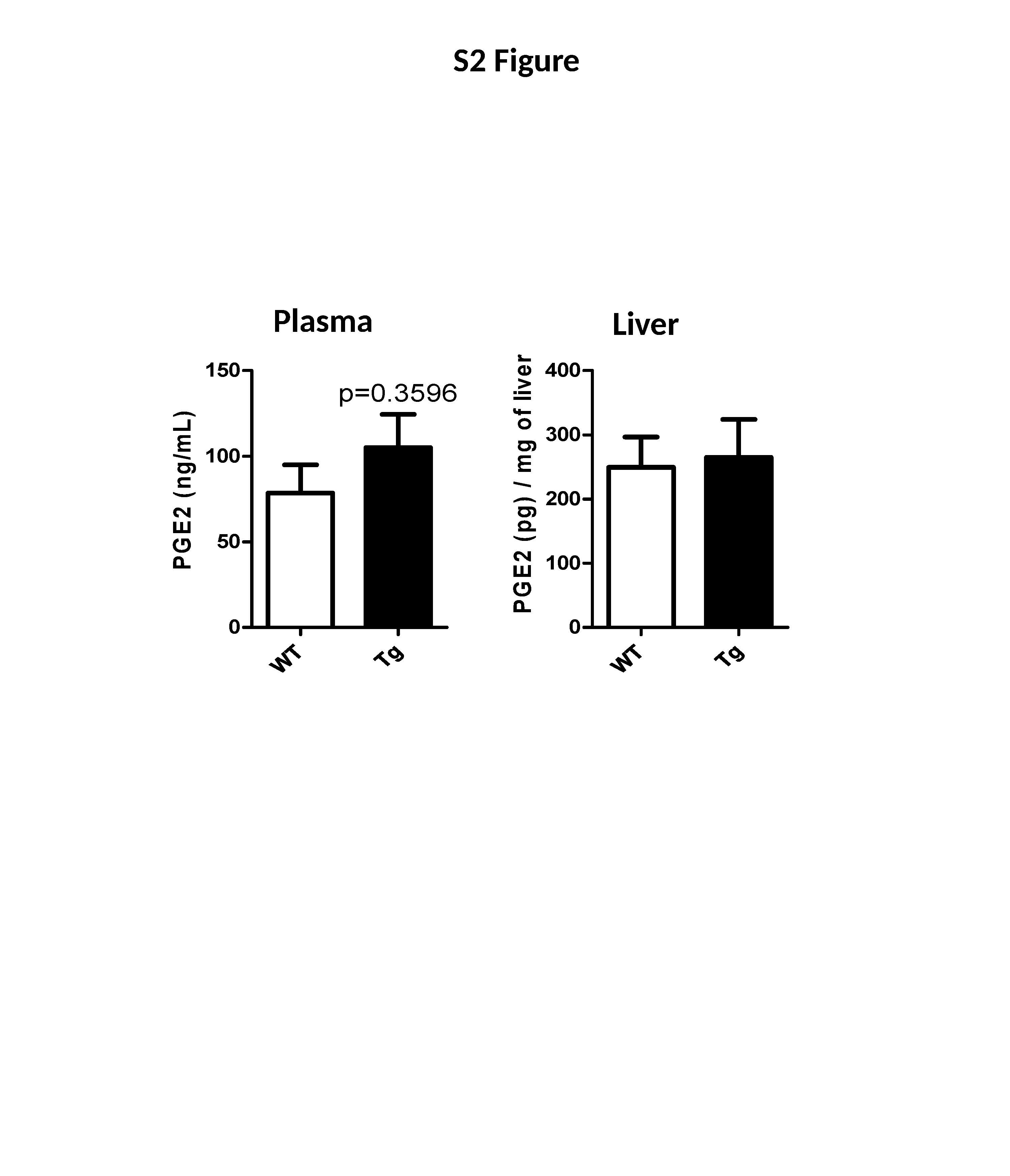

Supplement: S2 Fig — PGE2 levels were measured by Elisa as described in the Material and methods section, in the plasma and the liver of 1 year-old WT and Tg mice. The data are the means ± SEM of 3 mice per genotype. (TIF) [file pone.0130230.s002.tif]

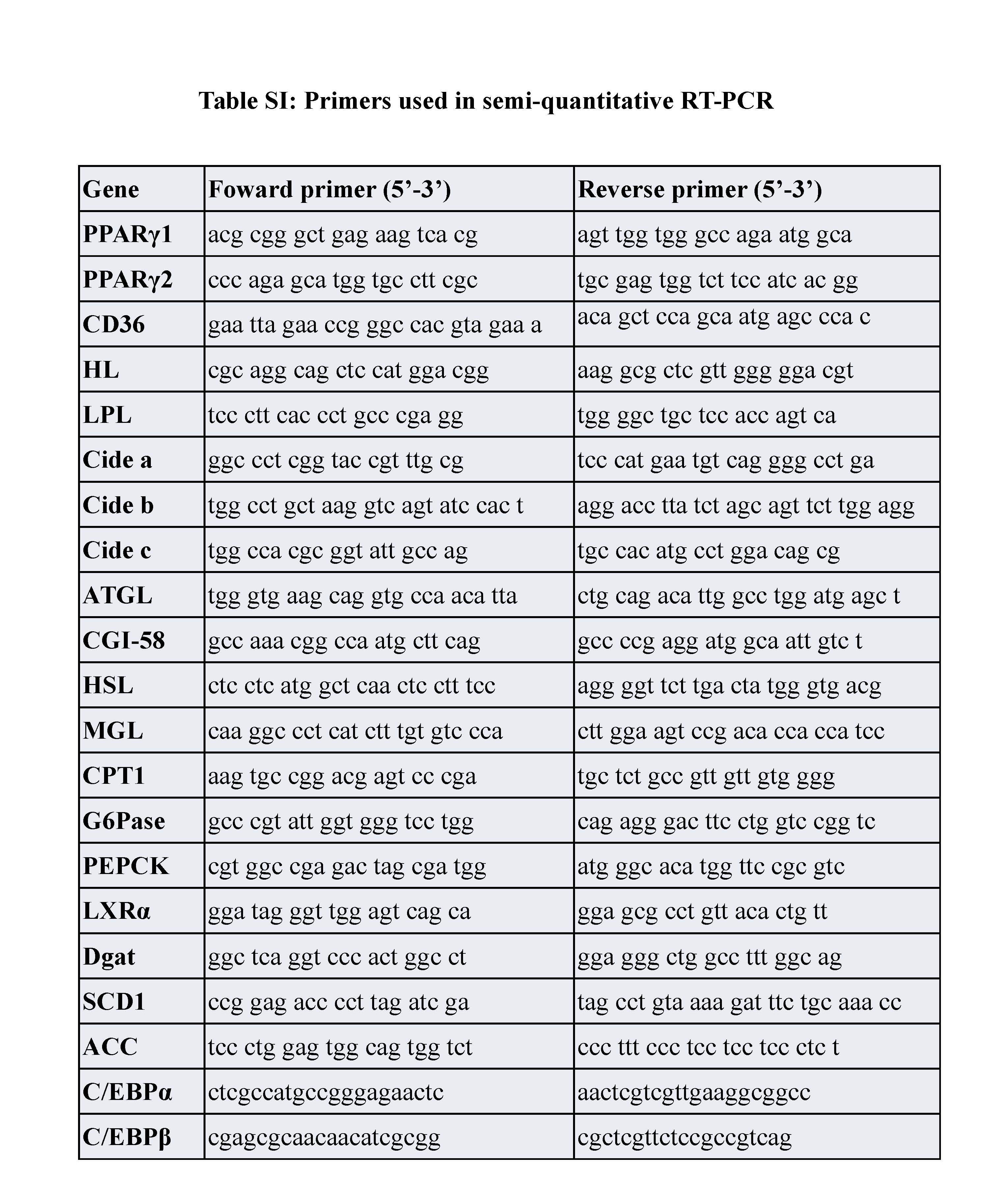

Supplement: S1 Table — The table indicates the sequence of the primers used in semi-quantitative RT-PCR analysis presented in the manuscript. (TIF) [file pone.0130230.s003.tif]
